# Safety or Risk? Exploring Perceptions of Firearm-Related Risks Among Military Service Members and Civilian Employees at a Military Installation

**Published:** 2025-07

**Authors:** Makala D. Carrington, Ian H. Stanley, Michael D. Anestis, Rachel L. Johnson, Jayna Moceri-Brooks, Craig J. Bryan, Megan L. Johnson, Justin C. Baker, AnnaBelle O. Bryan, Mengli Xiao, Marian E. Betz

**Affiliations:** ^*a*^ Department of Community and Behavioral Health, Colorado School of Public Health, University of Colorado An-schutz Medical Campus, Aurora, Colorado, USA.; ^*b*^ Firearm Injury Prevention Initiative and Center for COMBAT Research, Department of Emergency Medicine, School of Medicine, University of Colorado Anschutz Medical Campus, Aurora, Colorado, USA.; ^*c*^ New Jersey Gun Violence Research Center, Rutgers School of Public Health, Rutgers University, Piscataway, New Jersey, USA.; ^*d*^ Department of Biostatistics and Informatics, Colorado School of Public Health, University of Colorado Anschutz Medical Campus, Aurora, Colorado, USA.; ^*e*^ Department of Psychiatry and Behavioral Health, The Ohio State University College of Medicine, Columbus, Ohio, USA.; ^*f*^ VISN 2 Center of Excellence for Suicide Prevention, U.S. Department of Veterans Affairs, Canandaigua, New York, USA.; ^*g*^ Department of Emergency Medicine, School of Medicine, University of Colorado Anschutz Medical Campus, Firearm Injury Prevention Initiative, and VA Eastern Colorado Geriatric Research Education and Clinical Center, Aurora, Colorado, USA.

**Keywords:** Firearm injury, Military population, Firearm storage Practice, Firearm access, Firearm ownership

## Abstract

**Background:**

Firearm suicide ranks among the leading causes of death in the U.S. military, with access to personal firearms significantly elevating the risk of firearm-related injuries and death. In this study, we analyzed perceived risks of firearm access and storage among active-duty military service members and embedded civilians with a firearm at home.

**Methods:**

We conducted an anonymous online survey at a single military installation in the United States. Data were analyzed using logistic regression models across four firearm-related risk factors: suicide, others’ suicide, interpersonal violence, and unintentional shootings.

**Results:**

Of the 324 participants, 50.5% reported a minimum of one firearm at the home. Respondents with a minimum of one firearm at home (vs. those without) were less likely to agree that there was a risk of suicide for themselves (6.0% vs. 16.6%) or others (7.8% vs. 21.8%), interpersonal violence (16.4% vs. 26.9%), or unintentional shootings (27.9% vs. 42.3%). After adjusting for age, gender, race, ethnicity, and living alone, respondents with a firearm at home (vs. those without) were significantly less likely to agree that firearm access increased the risk of suicide for themselves (odds ratio [OR]: 0.20; 95% CI: 0.10, 0.40; p less than .001) or others (OR 0.19; 95% CI: 0.10, 0.36; p less than .001), interpersonal violence (OR: 0.25; 95% CI: 0.15, 0.43; p less than .001), or unintentional shootings (OR: 0.22; 95% CI: 0.13, 0.38; p less than .001).

**Conclusions:**

Our findings identify opportunities for strengthening messaging to help service members understand and acknowledge risks surrounding a firearm at home and promote secure firearm storage behaviors.

## Introduction

**Firearm Suicide Among Service Members: A Growing Concern**

Injury and death from a personal firearm have impacted military populations for decades.^1,2^ Over the years, firearm suicides have remained high across multiple branches of the United States (U.S.) Armed Forces.^3^ As a result, firearm suicide has become a pressing issue in the U.S. and its military.^4^ According to the Defense Suicide Prevention Office, 331 active-duty members died by suicide in 2022. Of all the suicides in 2022, 65% percent used a firearm, most commonly a personal rather than military-issued firearm, averaging approximately one suicide per day.^3^

Service members who own a firearm are at an increased risk of suicide than those who do not, due to the lethality of a firearm compared to other methods.^5^ Research indicates a strong connection between the prevalence of firearm ownership and access and the incidence of firearm-related suicides.^6^ A firearm in the home is linked to a fivefold increase in the risk of suicide, and in firearm-owning households, approximately 90% of suicides involve the use of a firearm.^6^ Cleveland et al. argued that due to significantly higher firearm ownership among veterans compared to the nation’s general population (49% for U.S. veterans and 22% for the general population), there is an increased risk of firearm injuries and deaths, including unintentional shootings, suicide, and interpersonal violence.^7^

**Perceived Risks and Firearm Ownership: Existing Literature**

Weinstein and Klein note that limited attention has been given to understanding whether service members perceive a firearm in the home as a risk to themselves and others.^8^ Survey respondents often underrate their vulnerability to risk while judging others who are “at-risk” as more susceptible, such as those with depression, substance abuse, or active suicidal thoughts. As a result, a cross-sectional analysis of the 2018 California Safety and Wellbeing Survey (n = 2558, 49% completion rate) explored safety-related beliefs associated with a firearm in the home.^9^ The study found that one in four respondents (regardless of ownership) stated that safety depended on firearm storage and access. Only 12% (4% of owners and 13% of non-owners) linked safety concerns to at-risk individuals (e.g., those with active suicidal thoughts). The factors that influenced safety beliefs included gun owner characteristics, firearm storage practices, and mental health. Findings showed that 15% of American adults believed that a firearm increased the risk of suicide and only 6% were firearm owners. Yet, secure storage (e.g., the firearm is stored unloaded, locked, and separated from ammunition) was less common in suicide attempts and unintentional injury cases, violence, and harm to others across military populations.^10,11^

The 'Protection Paradox,' a concept wherein new firearm owners, especially those who acquired firearms during the COVID-19 pandemic, perceive a reduction in risk despite facing increased actual danger, provides a relevant framework for understanding the complexities of firearm ownership.^12,13^ This paradox aligns with Buttrick's (2020) coping model of protective gun ownership, which posits that individuals adopt firearms as a psychological strategy to manage perceived threats to personal safety, control, and identity. Together, these models illuminate how the perception of firearms as protective tools may inversely affect individuals' risk assessments, setting a theoretical foundation for examining the nuanced perceptions of safety and risk among firearm owners.

Salhi et al. reviewed the 2019 National Firearms Survey and found that firearm owners' beliefs about whether a firearm makes a home safer have shifted since 2015. In 2015, firearm owners were primarily categorized into two groups: those who believed firearms made the home safer and those who believed they made it more dangerous; only a small percentage selected "it depends." By 2019, 34% of firearm owners chose "it depends," and authors reported a 40% increase in this response category. This shift suggests a growing awareness of the complexities of firearm-related risks as well as the need for further research into the factors influencing these ever-evolving perceptions.^14^ Barnett et al. acknowledged that key military leaders and academic research communities agree that a firearm in the home enables risk factors for suicide among former and current military members.^15^ Yet, there is limited research about the factors that affect service members’ perceived risks regarding a firearm in the home and whether those factors vary by gun ownership status or demographic variables. 

Moreover, Joe et al. underscore the significant role demographic variables such as age, gender, race, ethnicity, and having a firearm in the home play in understanding the perceived risks of firearm suicide.^16^ Demographic characteristics are not just superficial labels but are intrinsically linked to cultural, social, and psychological elements that shape an individual's interaction with firearms. Incorporating these demographic variables into the analysis is crucial for a nuanced understanding of firearm suicide risk, especially in specific populations like military personnel. It allows for the identification of targeted intervention strategies and the development of more effective, contextually relevant prevention programs. Several factors influence the perceived risks associated with a firearm at home; identifying these factors can guide customized messages and interventions for firearm injury prevention. 

**Addressing a Research Gap: Our Study's Objectives**

This study assessed the perceived risks of the presence of a firearm at home as well as storage practices among active-duty military personnel and civilians serving at a U.S. military installation. We hypothesized that service members and civilians with firearms in their homes, compared to those without firearms, would be less likely to perceive a firearm as increasing the risk of suicide, suicide by others, interpersonal violence, and unintentional shootings. These findings will help identify opportunities to strengthen messaging on the firearm-associated risks related to suicide, interpersonal violence, and unintentional shootings and promote secure storage practices across the military.

## Methods

This analysis used baseline, pre-intervention data from a more extensive program to implement a firearm injury prevention program at a military installation. At the time of the survey described herein (i.e., the pre-program survey), service members and embedded civilians (i.e., civilians working within a unit at a military installation alongside service members) had not received any intervention components.

**Study Population**

Eligible individuals were adult (18+) active-duty service members or embedded civilians at one Space Force Base that included both Air Force and Space Force members. The research team sent the survey to all Guardians and civilians working in the eight squadrons participating in the larger intervention program (approximately 862 personnel). This analysis included only those participants who answered the survey question asking if they had a personal firearm in or around their home. 

**Study Design**

The research team designed and emailed an anonymous survey (44 questions) to all eligible participants. Email invitations sent by a leader at the military installation included a hyperlink to complete the survey anonymously in a secure web-based data collection platform, REDCap.^17^ Eligible participants received three email reminders to complete the survey. Participants did not receive an incentive for survey completion, and participation was not mandatory. The survey included language acknowledging voluntary participation and the option to skip any questions. The Colorado Multiple Institutional Review Board and the Office of Human Research Oversight approved the project.

**Study Measures**

Firearm-related characteristics included the presence of a firearm in or around the home (“Do you have any personal firearms in or around your home”; “yes” or “no”), access to a firearm, and current storage practices. The survey included the following questions related to firearm access, “To what extent do you agree that having access to a firearm is related to the risk of…” for: “suicide for yourself?”; suicide for others in your home?”; “interpersonal violence?”; and “unintentional shootings?”. The survey also included questions related to firearm storage practices, such as, “To what extent do you agree that how a firearm is stored is related to risk for…” for the same four outcomes. These questions used a Likert scale for responses (Strongly Agree, Agree, Disagree, and Strongly Disagree). 

**Statistical Analysis**

Independent variables included having a firearm in the home, age, gender, race, ethnicity, and living alone. Dependent variables included perceived risk of suicide, suicide of others, interpersonal violence, and unintentional shootings related to firearm access, as well as perceived risk of suicide, suicide of others, interpersonal violence, and unintentional shootings related to firearm storage practices. Demographic variables were summarized with frequencies and percentages, with differences between groups tested with Fisher’s exact tests due to small numbers of participants in some demographic categories. Perceived risks associated with firearm access and storage practices were summarized with frequencies and percentages for Likert scale responses. Since several outcomes had small sample sizes within each Likert response, all were collapsed as agree (strongly agree, agree) vs. disagree (disagree, strongly disagree). Considering the 4-level variables, this re-coding was necessary to increase power due to many small categories. Re-coding each scale was also beneficial because it allowed the use of logistic regression for interpretability instead of ordinal regression for a four-level outcome.

Due to the small sample size, American Indian/Alaska Native (n = 4) and Native Hawaiian/Pacific Islander (n=2) groups were combined into one group, and non-binary (n = 2) participants were not included in the analysis cohort because collapsing across gender groups was not considered inclusive. All analyses were conducted in R version 4.2.1.^18^ Each outcome was modeled in a multiple logistic regression with Firth’s bias-reduced correction due to near or complete separation in some demographic variables using the “logistf” package.^19^ Additionally, we implemented adjustments for demographic variables in our analysis for two primary reasons: first, as evidenced in Table 1, demographic disparities are present in the patterns of firearm ownership, necessitating the control of these variables to isolate the impact of firearm possession. Secondly, our analysis aims to highlight the influence of each demographic factor on firearm ownership while concurrently controlling for the interplay of additional variables.

**Table 1:** Participant characteristics (N=324).

Demographic variable	N with data	Overall (N = 324)	Firearm in home (N = 166)	No firearm in home (N = 158)	p value
**Age**	**323**				**<0.001**
18-24		27 (8.4%)	3 (1.8%)	23 (14.6%)	
25-39		142 (44.0%)	73 (44.8%)	67 (42.7%)	
40-54		91 (28.2%)	56 (34.4%)	35 (22.3%)	
55+		63 (19.5%)	31 (19.0%)	32 (20.4%)	
**Gender**	**322**				**0.003**
Female		106 (32.9%)	42 (25.9%)	64 (40.8%)	
Male		214 (66.5%)	120 (74.1%)	91 (58.0%)	
Non-binary		2 (0.6%)	0 (0.0%)	2 (1.3%)	
**Race**	**318**				**0.673**
American Indian/Alaska Native		4 (1.3%)	2 (1.2%)	2 (1.3%)	
Asian		15 (4.7%)	5 (3.1%)	10 (6.5%)	
Black/African American		25 (7.9%)	11 (6.9%)	13 (8.4%)	
Native Hawaiian/Pacific Islander		2 (0.6%)	1 (0.6%)	0 (0.0%)	
White		243 (76.4%)	128 (80.0%)	114 (73.5%)	
More than one race		15 (4.7%)	6 (3.8%)	9 (5.8%)	
Other		14 (4.4%)	7 (4.4%)	7 (4.5%)	
**Ethnicity**	**313**				**0.600**
Hispanic/Latino/a		36 (11.5%)	17 (10.6%)	19 (12.6%)	
Not Hispanic/Latino/a		277 (88.5%)	143 (89.4%)	132 (87.4%)	
**Lives alone**	**321**				**<0.001**
Yes		61 (18.5%)	17 (10.2%)	44 (27.8%)	
No		260 (79.0%)	145 (87.3%)	113 (71.5%)	

## Results

The demographics of participants are summarized in Table 1. Of the sample of military service members and embedded civilians, five respondents were excluded because they did not answer the question, “Do you currently have any personal firearms in or around your home?”. Of all other respondents (N=324), 50.5% indicated having a firearm in the home. Respondents were primarily male (66.5%), White (76.4%), not Hispanic or Latino/a (88.5%), and living with at least one other person (79.0%). The most common age groups were 25-39 (44.0%) and 40-54 (28.2%). There were differences between those with and without a firearm at home in age, gender, and living situation. Respondents with a firearm at home were older on average, with only 1.8% in the 18-24 age group compared to 14.6% aged 18-24 in the participants without a firearm in the home (p <0.001). Participants with a firearm were also more likely to be male (74.1%) than those without a firearm in the home (58.0%) (p = 0.003) and less likely to live alone (10.2%) than those without a firearm (27.8%) (p<0.001).

Perceptions of risks associated with firearm access and storage are summarized in Table 2. Respondents who indicated having a personal firearm in or around their home (versus those who did not) were less likely to agree that there is a risk for suicide for themselves (6.0% vs. 16.6%, respectively), suicide for others in the home (7.8% vs. 21.8%), interpersonal violence (16.4% vs. 26.9%), and unintentional shootings (27.9% vs. 42.3%). Participants who indicated the presence of a firearm in the home (versus those who did not) were also less likely to agree there was risk related to firearm storage for suicide for themselves (9.1% vs. 21.9%, respectively), suicide for others in the home (14.5% vs. 21.8%), interpersonal violence (22.9% vs. 32.5%), and unintentional shootings (32.5% vs. 34.0%).

**Table 2:** Participant beliefs around firearm access, storage, and risk of suicide, interpersonal violence, or unintentional shootings, by the presence of a firearm in the home (N=329).

Outcome variables	N with data	Overall (N = 329)	Firearm in home (N = 166)	No firearm in home (N = 158)
**Access outcomes**				
**To what extent do you agree that having access to a firearm is related to risk of suicide for yourself?**	**328**			
Agree		36 (11.0%)	10 (6.0%)	26 (16.6%)
Disagree		62 (18.9%)	32 (19.3%)	30 (19.1%)	
**To what extent do you agree that having access to a firearm is related to risk for suicide for others in your home?**	**327**			
Agree		47 (14.4%)	13 (7.8%)	34 (21.8%)
Disagree		73 (22.3%)	38 (22.9%)	35 (22.4%)
**To what extent do you agree that having access to a firearm is related to risk for interpersonal violence?**	326			
Agree		69 (21.2%)	27 (16.4%)	42 (26.9%)
Disagree		61 (18.7%)	30 (18.2%)	31 (19.9%)
**To what extent do you agree that having access a firearm is related to risk for unintentional shootings?**	**326**			
Agree		112 (34.4%)	46 (27.9%)	66 (42.3%)
Disagree		51 (15.6%)	26 (15.8%)	25 (16.0%)
**Storage outcomes**				
**To what extent do you agree that how a firearm is stored is related to risk for suicide for yourself?**	**325**			
Agree		49 (15.1%)	15 (9.1%)	34 (21.9%)
Disagree		69 (21.2%)	35 (21.2%)	33 (21.3%)
**To what extent do you agree that how a firearm is stored is related to risk for suicide for others in your home? **	**327**			
Agree		59 (18.0%)	24 (14.5%)	34 (21.8%)
Disagree		66 (20.2%)	36 (21.7%)	30 (19.2%)
**To what extent do you agree that how a firearm is stored is related to risk for interpersonal violence? **	**328**			
Agree		90 (27.4%)	38 (22.9%)	51 (32.5%)
Disagree		67 (20.4%)	36 (21.7%)	31 (19.7%)
**To what extent do you agree that how a firearm is stored is related to risk for unintentional shootings? **	**327**			
Agree		109 (33.3%)	54 (32.5%)	53 (34.0%)
Disagree		38 (11.6%)	16 (9.6%)	22 (14.1%)

Each perceived risk outcome was modeled in a separate logistic regression that included all the following independent variables: having a firearm in the home, age, gender, race, ethnicity, and living alone. After adjusting for all demographics, those with a firearm in the home (versus those without) were less likely to agree that there was a risk for suicide for themselves (odds ratio [OR]: 0.20; 95% CI: 0.10, 0.40), suicide for others (OR: 0.19; 95% CI: 0.10, 0.36), interpersonal violence (OR: 0.25, 95% CI: 0.15, 0.43), and unintentional shootings (OR: 0.22; 95% CI: 0.13, 0.38) related to firearm access (Table 3). 

**Table 3:** Factors associated with belief that firearm access is related to risk of suicide, interpersonal violence, or unintentional shooting (N=324).

	To what extent do you agree that having access to a firearm is related to risk of:							
	Suicide for self		Suicide for others		Interpersonal violence		Unintentional shootings	
	Odds ratio (95% CI)	P value	Odds ratio (95% CI)	P value	Odds ratio (95% CI)	P value	Odds ratio (95% CI)	P value
**Firearm in home (ref: no firearm in home)**	0.20 (0.10, 0.40)	<0.001	0.19 (0.10, 0.36)	<0.001	0.25 (0.15, 0.43)	<0.001	0.22 (0.13, 0.38)	<0.001
**Age 25-39 (reference: 18-24)**	0.87 (0.30, 2.50)	0.795	1.11 (0.39, 3.18)	0.847	1.79 (0.67, 4.78)	0.241	0.66 (0.23, 1.88)	0.437
**Age 40-54 (reference: 18-24)**	1.45 (0.47, 4.49)	0.522	1.97 (0.64, 6.06)	0.231	1.80 (0.63, 5.14)	0.265	0.64 (0.22, 1.92)	0.428
**Age 55+ (reference: 18-24)**	1.33 (0.40, 4.38)	0.641	1.39 (0.43, 4.52)	0.585	2.01 (0.68, 5.96)	0.203	0.49 (0.16, 1.49)	0.204
**Male gender (reference: female)**	1.42 (0.75, 2.70)	0.283	1.75 (0.94, 3.27)	0.078	1.11 (0.65, 1.89)	0.700	1.41 (0.84, 2.36)	0.199
**AI/AN/NH/PI (ref: White)**	0.94 (0.12, 7.21)	0.951	0.72 (0.09, 5.62)	0.758	2.87 (0.51, 16.14)	0.238	1.23 (0.22, 6.85)	0.819
**Asian (ref: White)**	0.75 (0.18, 3.11)	0.684	1.00 (0.27, 3.64)	1	0.74 (0.23, 2.42)	0.618	0.92 (0.30, 2.84)	0.880
**Black/African American (ref: White)**	0.61 (0.18, 2.05)	0.410	0.92 (0.32, 2.66)	0.875	1.10 (0.44, 2.75)	0.834	1.23 (0.49, 3.08)	0.665
**More than one race (ref: White)**	1.41 (0.42, 4.80)	0.590	0.78 (0.21, 2.88)	0.709	1.68 (0.55, 5.17)	0.370	2.19 (0.59, 8.16)	0.233
**Other (ref: White)**	0.07 (0.004, 1.29)	0.018	0.18 (0.03, 1.17)	0.047	0.30 (0.06, 1.38)	0.101	0.26 (0.07, 1.02)	0.048
**Hispanic/Latino/a (ref: not Hispanic/Latino/a)**	2.59 (1.03, 6.52)	0.053	2.88 (1.17, 7.08)	0.026	1.66 (0.72, 3.81)	0.239	1.92 (0.79, 4.64)	0.146
**Lives alone (ref: does not)**	0.79 (0.37, 1.69)	0.548	0.83 (0.40, 1.71)	0.614	0.80 (0.42, 1.53)	0.496	0.51 (0.27, 0.97)	0.041

Demographic variables were included in the regression model as control variables to adjust for confounding. While adjusted odds ratios for these variables are presented, they should not be interpreted as direct causal effects on perceived risk. There were minimal effects of race on perceived risk, solely stemming from comparisons between White participants and participants who self-reported their race as "other," with those who reported their race as "other" being less likely to agree that there was a perceived risk of suicide for themselves or unintentional shootings compared to White participants. Finally, those who lived alone were less likely to perceive risks associated with firearm access related to unintentional shootings than those who did not live alone (OR: 0.51, 95% CI: 0.27, 0.97).

Similarly, after adjustment for demographic variables, those who indicated having a firearm in the home were less likely to perceive risks associated with firearm storage such as suicide for themselves (OR: 0.33; 95% CI: 0.19, 0.57); suicide for others (OR: 0.42; 95% CI: 0.25, 0.69), interpersonal violence (OR: 0.42, 95% CI: 0.26, 0.68), and unintentional shootings (OR: 0.42, 95% CI: 0.25, 0.71) than those who indicated they did not have a firearm in the home (Table 4). Finally, those who lived alone were less likely to perceive that unsecured firearm storage practices were associated with the risk for unintentional shootings than those who did not live alone (OR: 0.45, 95% CI: 0.24, 0.85). 

**Table 4:** Factors associated with belief that firearm storage is related to risk of suicide, interpersonal violence, or unintentional shooting (N=329).

	To what extent do you agree that having access to a firearm is related to risk of:							
	Suicide for self		Suicide for others		Interpersonal violence		Unintentional shootings	
	Odds ratio (95% CI)	P value	Odds ratio (95% CI)	P value	Odds ratio (95% CI)	P value	Odds ratio (95% CI)	P value
**Firearm in home (ref: no firearm in home)**	0.33 (0.19, 0.57)	<0.001	0.42 (0.25, 0.69)	0.001	0.42 (0.26, 0.68)	<0.001	0.42 (0.25, 0.71)	0.001
**Age 25-39 (reference: 18-24)**	0.92 (0.35, 2.41)	0.870	0.68 (0.27, 1.71)	0.414	0.76 (0.30, 1.92)	0.567	0.82 (0.29, 2.30)	0.704
**Age 40-54 (reference: 18-24)**	1.09 (0.39, 3.05)	0.874	0.73 (0.27, 1.96)	0.534	0.66 (0.25, 1.77)	0.412	0.71 (0.24, 2.12)	0.546
**Age 55+ (reference: 18-24)**	1.14 (0.39, 3.32)	0.805	0.99 (0.36, 2.74)	0.989	0.74 (0.27, 2.05)	0.563	0.66 (0.22, 2.01)	0.463
**Male gender (reference: female)**	1.06 (0.61, 1.83)	0.832	0.96 (0.58, 1.59)	0.863	0.87 (0.53, 1.43)	0.586	0.88 (0.52, 1.49)	0.635
**AI/AN/NH/PI (ref: White)**	0.85 (0.12, 6.07)	0.876	1.30 (0.24, 7.13)	0.764	16.12 (0.91, 286.89)	0.011	7.20 (0.40, 128.27)	0.092
**Asian (ref: White)**	1.09 (0.34, 3.54)	0.881	0.94 (0.31, 2.86)	0.914	1.51 (0.51, 4.47)	0.455	1.45 (0.42, 5.04)	0.556
**Black/African American (ref: White)**	1.72 (0.70, 4.22)	0.243	0.90 (0.37, 2.21)	0.822	1.09 (0.46, 2.59)	0.838	1.01 (0.41, 2.49)	0.990
**More than one race (ref: White)**	1.06 (0.32, 3.44)	0.929	0.70 (0.22, 2.24)	0.541	1.06 (0.36, 3.14)	0.918	2.28 (0.56, 9.31)	0.224
**Other (ref: White)**	0.81 (0.20, 3.32)	0.772	0.82 (0.22, 3.02)	0.760	0.57 (0.16, 2.10)	0.398	1.13 (0.30, 4.30)	0.857
**Hispanic/Latino/a (ref: not Hispanic/Latino/a)**	1.44 (0.62, 3.36)	0.411	1.39 (0.62, 3.10)	0.426	1.42 (0.64, 3.19)	0.395	0.86 (0.36, 2.03)	0.736
**Lives alone (ref: does not)**	1.06 (0.55, 2.04)	0.860	0.92 (0.49, 1.72)	0.798	0.68 (0.37, 1.27)	0.224	0.45 (0.24, 0.85)	0.015

## Discussion

This study found that respondents with a firearm in their home were less likely to acknowledge the risks related to firearm access and storage, such as suicide, harm to others, interpersonal violence, and unintentional shootings, compared to those without a firearm. Similarly, individuals living alone were less likely to perceive firearm access and storage as increasing the risk of unintentional shootings. These findings indicate a worrying lack of perceived risk among individuals, potentially leading to unsafe storage practices and increased injury, violence, and death in military households. Notably, there have been no reported suicide deaths for the Space Force as of the latest Department of Defense report.^3^ Many service members in the sample were unaware of or did not acknowledge the risks associated with unsecured firearm access in the home. Understanding these perceptions is crucial for developing effective firearm injury prevention strategies tailored to military populations.

**Implications**

This study underscores the need for enhanced messaging about the risks of firearm ownership, particularly addressing the "protection paradox," where individuals believe firearms make them safer despite evidence of increased risks.^12^ Given the links between firearm ownership and increased suicide and unintentional injury risks, promoting secure firearm storage is imperative.^11^ Public health interventions should universally advocate for secure firearm storage practices in military households to mitigate the risk of injury and death.

Furthermore, the research emphasizes the importance of understanding demographic influences on perceived risk. Future studies should investigate these disparities and tailor interventions accordingly. For example, examining perceived risk variations among service members from diverse racial and ethnic backgrounds within different military branches can inform culturally sensitive strategies.^8^

Researchers must recognize the diverse priorities and values across military installations, necessitating customized approaches. Engaging service members in discussions about risk reduction strategies and firearm storage practices is crucial for fostering acceptance and implementation of preventive measures.^20,21^ Such efforts between base leadership, service members, and researchers are essential steps in changing the perception of firearm-related risk and preserving the lives of those who serve our nation.^4^

**Limitations**

One limitation of this study is the presence of missing responses to specific survey items related to firearm-related risk perceptions. Half of the sample reported firearm ownership, but some participants may have chosen not to disclose this information. The nature of the questions, particularly those addressing perceived risks, may have influenced some participants’ willingness to respond. Additionally, firearm owners may have been less likely to report perceptions of risk, which could have impacted the observed differences between groups. The survey data also relied on self-reported data that may be influenced by social desirability bias or individual subjectivity. The study's single-site nature limits generalizability to other military populations and civilian settings. The causality between risk perception and firearm ownership cannot be determined due to the study's cross-sectional design, warranting longitudinal investigations for deeper insights. 

Participant anonymity was prioritized over detailed demographic data (i.e., precise age, rank) collection to ensure honest participation. Our data also does not specify the exact number or percentage of civilians versus military members or the number of individuals stationed or working on the base that participated in this study. Additionally, the survey did not inquire about firearms on military installations, a notable limitation given that most military firearm suicides involve personal firearms.^2^ Incorporating these details into future research could enrich the dataset, provided confidentiality is maintained.

Self-reported data and binary categorization of Likert scale responses simplified interpretation and improved model convergence due to small cell sizes but can compromise statistical power and increase the risk of Type I errors.^22,23,24^ Lastly, there is also the potential for misinterpretation of adjusted odds ratios for demographic variables. While these variables were included to account for confounding, their adjusted odds ratios should not be interpreted as causal effects.^25^ Future research could explore stratified models or other approaches to further investigate demographic factors separately.

## Conclusion

This study reveals that various factors influence perceived risks concerning a firearm being in the home, and in this sample, those who have a firearm perceived fewer risks on average than those without a firearm. Understanding and addressing these perceptions is crucial for developing effective, evidence-based firearm injury prevention strategies tailored to service members and civilian employees in military settings. Further research is needed to explore the nuances of perceived safety among service members across different demographics and to assess the effectiveness of targeted interventions and tailored messaging to promote safe firearm storage practices and reduce injuries and deaths associated with firearms across military populations.

**Authors’ Contributions:**

MDA, CJB, and MEB designed the overall study and secured project funding. MDC led study conceptualization and coordination; RLJ conducted the data analysis. MDC drafted the initial manuscript, and all authors contributed substantive edits. All authors reviewed and approved the final manuscript.
